# An Estimation of the Economic and Environmental Impact of Inhaler Devices Switch for Non-Clinical Reasons in COPD and Asthma: The Case for Spain

**DOI:** 10.3390/jmahp13030034

**Published:** 2025-07-17

**Authors:** Oriol Solà-Morales, Joan B Soriano, Míriam Solozabal-Coll, Jose Vicente Galindo

**Affiliations:** 1 Fundació HiTT, 08015 Barcelona, Spain; 2 Department Basic Sciences, Universitat Internacional de Catalunya, 08017 Barcelona, Spain; 3 Servicio de Neumología, Hospital Universitario de la Princesa, 28006 Madrid, Spain; 4 Facultad de Medicina, Universidad Autónoma de Madrid, 28049 Madrid, Spain; 5 Centro de Investigación Biomédica en Red de Enfermedades Respiratorias (CIBERES), Instituto de Salud Carlos III, 28029 Madrid, Spain; 6 Chiesi Spain, 08908 L’Hospitalet de Llobregat, Spain

**Keywords:** inhaler device switching, COPD adherence, asthma management, economic burden, environmental impact

## Abstract

In respiratory patients, limited adherence to and misuse of devices hinder the effectiveness of inhalation therapy. Switching inhalers for non-clinical reasons poses a risk of deterioration of respiratory disease and/or promotes poor adherence to therapy. The objective of this work was to explore the impact of device changes for non-clinical reasons on clinical outcomes (primary) and costs (secondary), including carbon emissions in Spain. After a comprehensive literature search, the increased use of resources following worsening outcomes was apportioned using Spanish cost data and following the recommended pathways for care. We calculated the cost of re-training these patients and attributed carbon emissions in metric tons of CO_2_ equivalent (tCO_2_eq) to the excess resource use. In Spain, the impact of uncontrolled switching for non-clinical reasons in COPD has an annual estimated cost of EUR 923/patient, leading to an excess annual expenditure of more than EUR 216 million. For asthma patients, the annual impact is almost EUR 263/patient, representing an additional EUR 118 million excess annual expenditure. The environmental consequence of both conditions can be equated to almost 45 thousand tCO_2_eq. Training all these patients on the new device would cost around EUR 35 million and would generate an extra impact reduction of about 2.6 thousand tCO_2_eq in carbon emissions levy.

## 1. Introduction

The first pressurized metered dose inhaler (pMDI) was introduced in the market in 1956 [1]. Treatment of respiratory patients would hardly be imaginable without inhalers, as they are used in many different therapeutic areas, including chronic obstructive pulmonary disease (COPD) and asthma, the two most prevalent respiratory diseases. According to the Institute for Health Metrics and Evaluation, in 2019 alone, chronic respiratory diseases (the definition includes some other less prevalent diseases also using inhalers) were the third leading cause of death responsible for 4.0 million deaths, with a prevalence of 454.6 million cases globally [2].

Recent research on respiratory mechanisms has also led to the flourishing of new pharmacological solutions that enable the appropriate management of hard-to-treat patients. The loss of patents and the abundance of alternatives have led to a commoditization of respiratory therapies, and in some countries to a race-to-the-bottom drug cost pathway touting the loss of perceived value of these therapies [3], as respiratory care is a major source of financial stress to most healthcare systems. In just the EU28 [4], the direct respiratory drugs cost was EUR 42.8 billion in 2014, and is steadily growing. Furthermore, their environmental impact is forecasted to increase in healthcare, as shown by Planetary Medicine [5] theory. In Australia, where the damage of the bushfires [6] is costly in terms of lives lost and cardiovascular care costs, temperature-related respiratory hospitalization costs [7] are expected to increase 65–70% by 2050. The increase in respiratory diseases will increase the use of respiratory medicines.

Recently, there has also been a perception that inhalers have been under scrutiny due to the impact they can have in terms of carbon emissions, as the propellants used in pMDIs are identified as greenhouse gases. In fact, this scrutiny has evolved over the years. From the transition of chlorofluorocarbons (CFCs) to the current hydrofluorocarbons (HFCs) propellants in response to the Montreal Protocol, with the aim of protecting the ozone layer, to the recent revision of European regulation on fluorinated gases in order to align and comply with the mandate of the Kigali Amendment to reduce the production and consumption of HFCs, greenhouse gases significantly contribute to global warming [8].

The debate on the potential impact of inhalers on the environment is not only fair but also relevant and needed, and it is important to understand their complete picture. In Spain in July 2022, the Spanish Health Agency (AEMPS), in line with the current Spanish guideline GEMA 5.4. [9,10], indicates that until the arrival of new low global warming potential propellants, dry power inhalers (DPIs) may be used in new patients, if appropriate according to patient characteristics and their preferences [11]. In February 2025, the Spanish Ministry of Health (MoH) also published recommendations on the “Sustainable prescription of inhalers” [12], where they indicate that the prescription of inhalers with a lower carbon footprint (DPI, SMI, or other devices with low carbon footprint that may appear in the future) should be prioritized for new cases if the age, characteristics, and clinical characteristics of the patient allow it. The MoH added that, in general, it does not recommend changing inhalers due to environmental reasons in clinically controlled patients; adherence to treatment and the correct use of inhalation technique are essential for the respiratory diseases’ control. So, neither can all patients use DPIs, nor can they be recommended under certain circumstances. Overall, DPIs account roughly for 47% of the inhaled market [13], although the percentage rises to about 70% when looking only at chronic treatment. This indicates that almost 70% of prescribed pMDIs are rescue inhalers (Short-Acting Beta Agonists, SABA) (IQVIA data). Indeed, the Spanish guideline GEMA 5.4 and the MoH also stress the importance of appropriate prescription of chronic inhalers to try to keep the patient controlled and to reduce the excessive use of “rescue inhalers on demand” (SABA) to avoid excessive pMDI dosing and usage, all activities with high environmental impact.

It is also significant to underscore the fact that an important proportion of the climate impact of inhalers is not due to the usage of the drug, but rather to its misuse. Limited adherence (50% in COPD, 30–70% in asthma) and misuse (>50%) of devices hinder the effectiveness in inhaled therapy, thus leading to excessive rescue medication, which on many occasions is only available in pMDIs. Choosing the right device for each patient and enforcing patient education are key to keep patients with diseases under control, improving inhaled technique and adherence to treatments.

The MoH [14,15], the AEMPS [11], and the Spanish guideline GEMA 5.4 [9], among other publications [2,16], also documented that switching inhalers for non-clinical reasons often poses a risk of deterioration of the disease and/or increases poor adherence to therapy. However, there has been very little attention to the cost and environmental impact of switching devices for non-clinical reasons. It has been proposed that the lack of appropriate management of new devices following changes of therapy and the lack of device-management education leads to exacerbations per se. The argument is that when patients are asked to change their inhaler, their inhalation technique is likely to be poor thus leading to exacerbations. A real-world data estimation [17] suggests 9% of respiratory exacerbations are due to switching devices, but others have increased this estimate up to 16% in three EU countries [18].

Following from these estimations, we explored the impact of such unsolicited or not appropriately trained switching, to calculate their impact on health (primary objective), budget, and the environment in terms of carbon emissions generated (secondary objectives). Thus, we aimed to determine the impact of device switching for non-clinical reasons on health outcomes and cost, including carbon footprint in Spain.

## 2. Materials and Methods

To determine the health and economic impacts, including those on the environment in terms of carbon emissions of the inappropriate switching of inhalers, we built a forecasting consequentialist model to understand how those switches would impact healthcare. For simplicity, we based our model on the recommendations by SEPAR (Spanish Society of Pneumology and Thoracic Surgery) which do relate or mimic the recommendations of the European Respiratory Society, which are updated regularly from the international GINA [19] and GOLD [20] guidelines. The model was limited to both asthma and COPD to make it manageable but would certainly benefit from other extensions to other respiratory diseases. The model focuses mainly on the adult population for COPD and asthma, but also includes data from pediatric population in asthma. From guidelines, we obtained the expected follow-up recommendations, such as the expected healthcare visits following admissions or exacerbations.

The model considers prevalence of both diseases and then uses the published literature on severity (moderate and severe) to estimate those patients that are at risk of exacerbations. These exacerbations are then calculated, and the number of hospitalizations is also inferred, leading to an absolute number of admissions to hospital, emergency visits, and GP consultations. The number of exacerbations in mild patients is also calculated, but the assumption is that these are managed in the outpatient setting.

We then estimated the economic and environmental costs of these exacerbations, and what would be the annual cost to prevent them. We have used official data from the Spanish MoH [21] and its National Statistics Institute [22] to estimate the average hospital stay, costs, and epidemiology. Data shown reflect annual costs. The target population was those 40 years of age and older for COPD, and for asthma we also considered both pediatric (0–12 years) and adult populations.

We obtained from the literature the average levy in carbon footprint for an average hospitalization (bed day) and a GP visit assuming patients would have to travel there (we did not consider telemonitoring and/or remote visits). We then estimated the proportion of exacerbations that are directly due to device switches for non-clinical reasons, hence leading to the proportion of COPD and asthma exacerbations that are due to such device switches. In 2023, Sangiorgi et al. [17] published an Italian retrospective study based on the review of 2 M patients, where they quantified the risk of exacerbation after an inhaler switch; following this publication, we will only consider 9% of the population as a conservative approach and as a trend, though it is arguable that all patients should be regularly re-trained as the frequency of exacerbations due to improper device knowledge is uncertain. These and other authors suggest the link between poor inhalation technique, switching to a not well-managed device and the risk for exacerbation [14,15]. We have assumed hospitalized patients do not have enough inhalation capacity and hence the impact of the device is limited, as this is adapted to their capacity.

And finally, we estimated the impact attributable to switchers and the additional cost it would take the healthcare system to train those who are prescribed a switch of inhaler, considering that a prescription would trigger an ambulatory care visit (most likely to a nurse) and that this would incur a carbon footprint increase. We did not consider additional impact of wastage because data are lacking in individual costs and the costs used are aggregated care costs.

## 3. Results

The latest prevalence estimates for COPD and asthma in Spain are 11.80% and 7.23% (weighted average of pediatric −15%—and adult −5%) of the population, respectively, of which about half refer to moderate and severe cases, and of which one in five to seven are admitted to the hospital annually (Table 1). According to the Spanish Ministry of Health, the average cost of a COPD hospitalization is 2.2-fold more expensive than an asthma hospitalization. In January 2022, the estimated population in Spain was 47.4 M inhabitants, of which 49% were 40 years or older. Also, according to literature [23], a hospital bed-day footprint can be estimated as 35.53 Kg CO_2_ equivalent, and a GP visit can be estimated as 4.8 Kg CO_2_ equivalent [24].

For COPD (Table 2), we estimated 631,458 hospital admissions per year and almost 14 million visits to the GP because of exacerbations. Their overall cost is estimated to be EUR 2.36 billion in admissions and almost EUR 40 million in GP visits. For asthma, the impact to the healthcare system because of hospital admissions and GP visits is more than EUR 1.31 billion.

**Figure 1:**
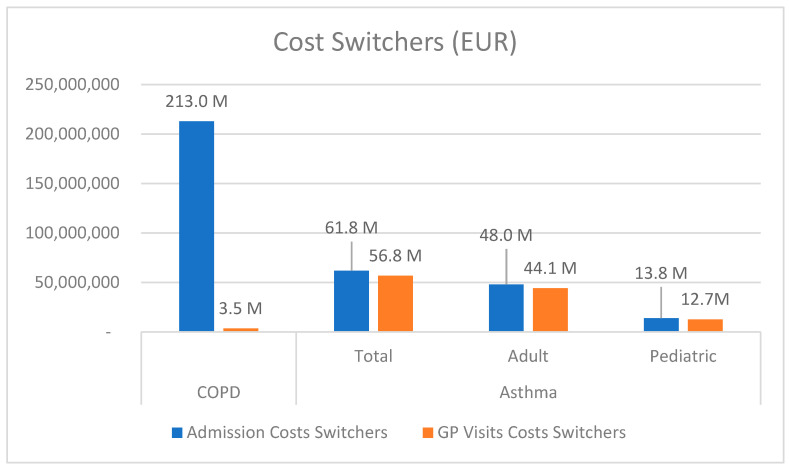
Source of costs for inhaler switchers for non-clinical reasons per year.

Assuming that 9% of these exacerbations are due to the switching of devices for non-clinical reasons, we estimated that about 57 thousand admissions in COPD and more than 36 thousand asthma admissions are directly linked to this switch, leading to a total cost of EUR 335 million per year (EUR 216 million because of COPD) (Table 2). Of note (Figure 1), in asthma the expenditure is shared between admissions and GP visits, whereas it is manly a hospital cost for COPD.

The number of admissions is remarkable in both COPD and asthma. An incidental changing of inhaler (switcher) is likely to impact patients’ health prospects, could lead to about 57 thousand and 36 thousand admissions for patients, leading to resource consumption, reduction in quality of life, and impact on their caregivers.

**Figure 2:**
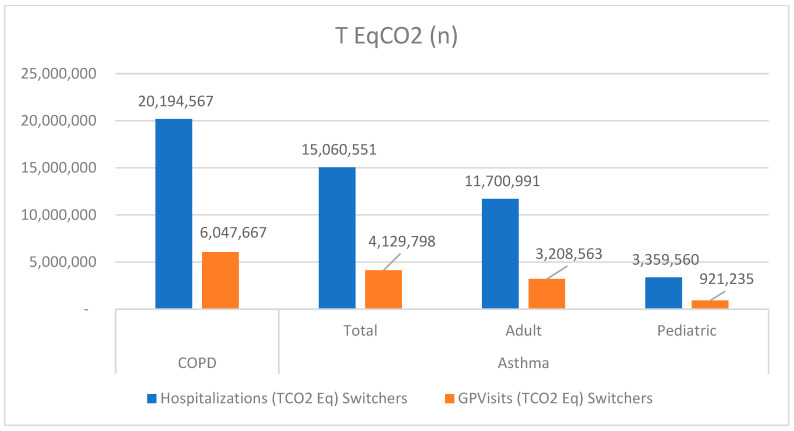
CO_2_ Footprint equivalent tons for COPD and asthma inhaler switchers for non-clinical.

Moreover, both hospital admissions and GP visits have a carbon footprint (Table 3 and Figure 2) due to inappropriate switching, amounting to more than 45 thousand tons of CO_2_ equivalent (tCO_2_eq) per year, but in this case mainly as a consequence of hospital stay in adults.

Finally, re-training all these moderate-to-severe exacerbated patients would require almost 0.5 million visits to the GP for COPD or asthma (Table 4), with an environmental impact equivalent to more than 2 thousand direct tCO_2_eq. To re-train these patients, a minimum of 114 professionals would be needed.

## 4. Discussion

The impact on healthcare of switching devices for non-clinical reasons is massive and under-recognized. Just for COPD and asthma adapted for the Spanish scenario (about 10.5% of the EU population), the cost of these switchers is about EUR 335 million per year. And the environmental cost is beyond 45,000 tCO_2_ equivalent, which is by no means an insignificant figure, especially when considering these are annual figures.

The bulk of the debate in respiratory medicine when discussing costs is on adherence [2] to treatment, the increasing awareness on the importance of training on device utilization [31], the sustained harm of tobacco [32], and gender imbalance [33]. In recent years, unsurprisingly, we have seen an increasing number of publications focusing on the climate change impact and associated challenges for health and respiratory medicine. Important associations have already been identified [34,35] with pollution, increased temperatures, and longer pollen exposure or natural disasters. Possibly as a consequence of this increased interest, environmental epidemiologists have highlighted the impact of pMDIs on carbon emissions and this has captured the interest of the general media, decision-makers, and researchers [36], who are rightly pushing for a fast-track switch to less environmentally concerning and equally effective DPIs.

However, the debate is flawed by two main reasons: not all patients can use DPIs and there are unintended side effects of switching for non-clinical reasons, which is the main focus of this publication. What seems certain is that changes need to be implemented, as missing a solution could lead to negative consequences.

It is a well-known fact by pulmonologists and GPs that not all patients can use DPIs. A minimum of 30 L/min flow inspiratory rate is a prerequisite for using DPIs [37] effectively and certain inhalation skills are required, that can and should be trained and maintained to obtain the necessary effect. Minimum literacy capacities are also needed [38], age differences are to be accounted for [15], and the multiplicity of devices is known to be confusing [39,40]. However, there is a limited perception that, especially during respiratory exacerbations, patients (generally induced by their treating physician) may change devices, leading to misuse and to switching-related exacerbations. Because of the former arguments (age, capacity, literacy, flow rate, …), respiratory patients may need extra training and education before these changes. Sangiorgi et al. [17] suggested that 9% of the exacerbations are directly attributable to inappropriate device changes, but others [18] have estimated it led to as much as 15.83% of hospitalizations.

Building on this idea, we estimated the impact of unplanned changes in inhaler devices, taking into account the recommendations in treatment guidelines (international and national) to raise the awareness of the importance for clinicians and the healthcare services to appropriately direct and manage the appropriate device fit in every circumstance. By no means do we suggest that DPIs should not be used when appropriate, but non-clinical changes lead to a massive impact to patients and the healthcare system, and ultimately to the environment. An excess of hospitalizations and GP visits is an unnecessary side effect of inappropriate device change, revealing the hidden part of the iceberg. But also very important, because these patients will incur additional treatments (as rescue inhalers), hospitalizations, and will need to travel to their GP appointments as a consequence of the mismanagement of inhaler devices, they will also incur significant costs and a higher carbon footprint.

As much as we believe action needs to be taken, we also wanted to highlight the importance of doing it at a pace that is manageable for the healthcare system. It should be noted that only one-third of the patients receiving maintenance therapy for COPD could be considered as having the disease controlled [41], and a non-staggered approach may lead to worse outcomes. From a policy perspective, this approach raises concerns not only due to the largely unbearable cost, but especially because of the health-related consequences for patients, who would be unable to manage exacerbations on their own and could be forced into hospital admission.

All in all, we need to be cautious in any transition, as we do not want the solution to be part of the problem. For instance, the improvement of treatment adherence could lead to better patient control, as it is a related factor [41], reducing the number of exacerbations and the consequent use of resources and carbon footprint associated. While inhalers have different carbon footprints, the best possible inhaler is the one that contributes to achieving optimal disease control, improves patient adherence, and minimizes side effects [12]. Furthermore, the transition to propellants with lower global warming potential (GWP) for pMDIs, that will reduce by up to 90% the carbon footprint of current pMDI products compared to current ones, and will be in the same range as the DPI carbon footprint [42,43,44,45], will be a good solution to reduce the current impact while maintaining patient needs, preferences, and access to all devices.

This research has some embedded limitations. First, calculations are based on projections from the general population, but it does not consider that some patients may have good management of several devices. There is some work suggesting this could be the case [46], though these analyses have limited scope and conflict with the widespread evidence that patients become confused when having to manage different medications in different devices. The figures we present represent the Spanish epidemiology, and adaptations to other geographies would require local clinical and cost data. It is also important to note that we have used 9% as a baseline figure, though there are publications pointing to estimates almost twice as high. We would not like to focus as much on the figure but on the trend, which is therefore very consistent and clear. Similarly, when calculating the environmental impact, we have not assessed the cost and consequences of recycling plastics, parts of the devices, and anything else, which is obviously of concern. All should lead to a more thoughtful decision when suggesting patients to change devices. The data used come from a rather old database, but we still think the data are fully valid for our initial assumptions. Finally, we need to understand that calculations of carbon footprint are in their infancy. There are very limited publications on the amount of carbon equivalents that are generated on a bed-day stay or the impact of going to the GP for checks, let alone the fact that some may use public transportation (how does one account for that when it is already running and one may or may not use it), or that different patients may be cared for in different complexity care settings (with or without ambulance, specialized wards, …). To our knowledge, there are no reliable calculations that delve into these details.

## 5. Conclusions

Switching from a device to another is sometimes unavoidable given the clinical condition of patients, and the healthcare system should train all of the switching population in order to avoid some of the known side effects of switching. But uncontrolled switching for non-clinical reasons should be avoided as it may result in a much higher clinical, economic, and environmental impact than the current situation.

There are other current and future solutions that could also lead to a reduction in the carbon footprint of the inhaled products without a risk of patient lack of control and maintain their needs and choices.

## Figures and Tables

**Table 1:** Epidemiology and cost of COPD and asthma.

	COPD	Asthma
Prevalence	11.80 [25]	7.23 [26]
% moderate and severe	53.10 [27]	49.00 [27]
Annual exacerbations (n)	2	1.86
% exacerbations admitted (hospitalizations)	21.5% [28]	13.0% [29]
Length of stay (days) [21]	8.48	5.54
Average cost per admission (EUR) [21]	3748	1691
GP visits per episode *	2.53	1.5
Average cost per GP Visit (EUR) [30]	66	

* Based on SEPAR’s CPG.

**Table 2:** Health and economic impact of COPD and asthma and the impact of switching inhalers for non-clinical reasons per year.

	COPD	Asthma
Admissions (n)	631,458	405,969
Cost of Admissions (EUR)	2,366,801,803	686,567,269
GP Visits (n)	13,999,228	9,559,718
GP Cost (EUR)	39,150,384	630,941,398
Average Combined Cost x Patient (EUR)	10,265.66	2919.87
**Total Cost (EUR)**	**2,405,952,187**	**1,317,508,666**
**9% of the Exacerbations are due to Switchers**		
Admissions (n)	56,831	36,537
Cost Admissions (EUR)	213,012,162	61,791,054
GP Visits (n)	1,259,931	630,941,398
Cost GP Visits (EUR)	3,523,535	56,784,726
Average Combined Cost x Switching Patient (€)	923.91	262.79
**Total Cost Switching Patients (EUR)**	**216,535,697**	**118,575,780**

**Table 3:** CO_2_ footprint equivalent tons for COPD and asthma inhaler switchers per year.

	COPD	Asthma
Admissions (Kg CO_2_eq)	20,194,567	15,060,551
ED Visits (Kg CO_2_eq) *	272,790	175,379
GP Visits (Kg CO_2_eq)	6,047,667	4,129,798
**Total (tCO_2_eq)**	26,242	19,190

* Calculated as every severe exacerbation leads to one ED visit; each visit equivalent as traveling to the GP. Visits to the GP or ED for non-severe episodes are not taken into account in the calculations. ED: Emergency Department.

**Table 4:** Cost of re-training the exacerbated population in COPD and asthma in switchers.

	COPD	Asthma	Total
number of exacerbations (GP Visits)	264,332	281,056	**545,387**
GP Visit cost (EUR)	17,445,881	18,549,677	**35,995,558**
tCO_2_eq	1269	1349	**2618**

## Data Availability

The original contributions presented in this study are included in the article. Further inquiries can be directed to the corresponding authors.
